# Neutral Science

**Published:** 1889-05-18

**Authors:** 


### The Hospital

A Weekly Institutional Journal of

**Science, Medicine, Nursing, and Philanthropy.**

*For Hospitals, Asylums, Medical Practitioners, Students, Nurses, Families, Parishes, Congregations, and the Charitable Public.*

Vol. VI. No. 138.] [May 18, 1889.

### Neutral Science.

The recent conversazione of the Royal Society reminds us of the existence of that body and of its doings among the children of men. The large number of variously distinguished persons who thronged the society's rooms the other evening were sufficient to convince the most sceptical that the star of science in these times is conspicuously in the ascendant. Rank, wealth, learning, politics, religion, and medicine all conspired to cry "hail" to the occupant of the president's chair in his capacity of representative worker in scientific fields. It was a noteworthy circumstance.

The search after knowledge has become a widespread passion of late years. The chief reason for this may be that knowledge is one of the surest, if not one of the most difficult, avenues to distinction. That it is not one of the most difficult is shown by the fact that a very considerable number of the more recently created fellows of the Royal Society are comparatively unknown men who have given evidence of no particular capacity, except such as is required for the, by no means very intellectual, work of physiological or other kind of original research. To the uninitiated the words "original research" have a very learned sound, and seem to imply much. But nobody knows better than the really intelligent fellows of the Royal Society, that they do not necessarily imply more than special opportunities, average mental capacity, and respectable industry. The ordinary man of science, so far as regards his general mental endowment, is on a level with the ordinary parson, doctor, or other educated man. The general public are not yet quite aware of this fact, and therefore are accustomed to attach an exaggerated importance to all that men of science say and do. In due time it will come to be understood that the conscientious scientific man knows his specialty exactly as the conscientious doctor knows physic, and that his opinions on general questions are of no more added value on account of his science than are the opinions of the doctor on account of his medicine. "Every man to his trade" is as good a rule for scientific men as for persons of every other class.

The tone of mind which characterises the members of the scientific world at the present moment is of a somewhat neutral character. "After a storm a calm." Scientists have been fighting a very aggressive battle for many years, and their final victory is of a decided and brilliant character. Like other victors, they are minded for the present to enjoy the spoils. Having conquered widespread recognition, they are content to sheath their swords. But it may not be improper to remind them that it is one thing to conquer recognition for themselves, but quite another to conquer the world for their sciences. The really heroic figures in the scientific world have seldom been men of the laboratory alone. They have mostly had something of the apostle in them Carlyle held that a man strengthened his own convictions immensely by imparting them to others and converting the others to his own side. Prof. Huxley, in the recent little bit of autobiography he gave to the public, declared, what everybody already knew, that he had always had a passion for popularising his scientific knowledge; and that he had been quite content that his mere academic fame should run the risk of losing some of its lustre, if he could only ensure the acceptance of scientific principles and methods over a large popular area. As a matter of fact, his academic fame has not only not been dimmed, but has rather been made more brilliant by the line of conduct he marked out for himself, and to which, to this day, he has so loyally adhered. Huxley is made of genuinely apostolic stuff, however surprising that may seem to some. He has deep and intelligent convictions, and he knows their value to the world. Like a true apostle, he can by no means be content until he has made every worthy and intelligent mind see the things of science as he sees them. With some of his published theological conclusions we have no kind of agreement whatever. In our judgment, if he fails anywhere, it is in that large region of human passions, fears, hopes, and speculations upon which religion has laid her hand. He is hard and literal. The poet seems to have been left out of his composition. He understands things better than men, "somites" better than souls. That is his peculiar limitation. But as an expounder of the principles and methods of his own department of physical science, Prof. Huxley has no living superior, if, indeed, since Clifford's death, he has an equal.

It is the misfortune of science and an irreparable loss to the world that men of Huxley's stamp are so exceedingly limited in number. Not one in a thousand has his powers of exposition. Many men of science, even if they had Huxley's unrivalled capacity for setting forth facts and principles, manifest no disposition whatever to shed the light of their scientific knowledge on a world that intellectually starves and dies for lack of such wisdom. Perhaps it is the consciousness of inferior gifts that makes them shrink from comparison with a master, and that prompts them to speak with disparagement of powers which they do not possess. Huxley's specialty has never been "human physiology"; and yet it is to him we owe the only worthy attempt that has ever been made to popularise that most interesting, important, and educationally valuable science. The medical professors of physiology are numbered by the score, perhaps by the hundred, among English-speaking peoples. Which of them has had the philosophic catholicity of mind even to desire to make the facts of his science the common possession of all intelligent men ? Can it be possible that these scientific professors really believe physiology to be the exclusive property of the medical profession, and that all other human beings are bound to be content with the scraps and crumbs which from time to time may casually fall from the medical table ? It is impossible to think otherwise than that some some such utterly childish superstition is in possession of the medical mind.

The scientific world, with a few brilliant exceptions, lacks enthusiasm. It is too redolent of the workshop. The man who merely plods in the laboratory is a very third-rate scientist. He is like the painter who mixes his colours well, but dare not touch a canvas for fear of spoiling it. It is a poor conception of science that merely observes and experiments. What is needed to convert knowledge into science is the philosophic mind that sees every fact in its true relation, and all the facts in their general bearing on the universal cosmos. If scientific men would feel it their duty to make all the world understand their work and its bearing upon all science and all life, the mere effort to expound their facts to the unlearned would both clear their own mental vision and mightily improve their intellectual capacity. The cold-blooded neutrality of many members of the scientific world is morally disgraceful, and intellectually ridiculous. Moses, a great leader, devoutly wished that all the children of Israel were prophets. It would be the dawn of a brighter day for the world than it has for a long time seen, if all the men of science were Huxleys.

